# Docosahexaenoic Acid Supplementation during Pregnancy: A Potential Tool to Prevent Membrane Rupture and Preterm Labor

**DOI:** 10.3390/ijms15058024

**Published:** 2014-05-07

**Authors:** Emanuela Pietrantoni, Federica Del Chierico, Giuliano Rigon, Pamela Vernocchi, Guglielmo Salvatori, Melania Manco, Fabrizio Signore, Lorenza Putignani

**Affiliations:** 1 Department of Obstetrics and Gynaecology, San Camillo Forlanini Hospital, Circonvallazione Gianicolense, 87, Rome 00151, Italy; E-Mails: anguela@libero.it (E.P.); rigon.giuliano@gmail.com (G.R.); fsignore@scamilloforlanini.rm.it (F.S.); 2 Unit of Metagenomics, Bambino Gesù Children’s Hospital, IRCCS, Piazza Sant’Onofrio, 4, Rome 00165, Italy; E-Mails: federica.delchierico@opbg.net (F.D.C.); pamela.vernocchi@opbg.net (P.V.); 3 Interdepartmental Centre for Industrial Research-CIRI-AGRIFOOD, Alma Mater Studiorum, University of Bologna, Piazza Goidanich, 60, Cesena-FC 47521, Italy; 4 Unit of Neonatology, Bambino Gesù Children’s Hospital, IRCCS, Piazza Sant’Onofrio, 4, Rome 00165, Italy; E-Mail: guglielmo.salvatori@opbg.net; 5 Scientific Directorate, Research Unit for Multifactorial Disease, Bambino Gesù Children’s Hospital, IRCCS, Piazza Sant’Onofrio, 4, Rome 00165, Italy; E-Mail: melania.manco@opbg.net; 6 Unit of Parasitology, Bambino Gesù Children’s Hospital, IRCCS, Piazza Sant’Onofrio, 4, Rome 00165, Italy

**Keywords:** pregnancy, maternal and fetal health, fetal growth, placental disorders, premature rupture of membranes (PROM), preterm-premature rupture of membranes (pPROM), DHA supplementation

## Abstract

Polyunsaturated fatty acids (PUFAs) are required to maintain the fluidity, permeability and integrity of cell membranes. Maternal dietary supplementation with ω-3 PUFAs during pregnancy has beneficial effects, including increased gestational length and reduced risk of pregnancy complications. Significant amounts of ω-3 docosahexaenoic acid (DHA) are transferred from maternal to fetal blood, hence ensuring high levels of DHA in the placenta and fetal bloodstream and tissues. Fetal DHA demand increases exponentially with gestational age, especially in the third trimester, due to fetal development. According to the World Health Organization (WHO) and the Food and Agriculture Organization of the United Nations (FAO), a daily intake of DHA is recommended during pregnancy. Omega-3 PUFAs are involved in several anti-inflammatory, pro-resolving and anti-oxidative pathways. Several placental disorders, such as intrauterine growth restriction, premature rupture of membranes (PROM) and preterm-PROM (pPROM), are associated with placental inflammation and oxidative stress. This pilot study reports on a preliminary evaluation of the significance of the daily DHA administration on PROM and pPROM events in healthy pregnant women. Further extensive clinical trials will be necessary to fully elucidate the correlation between DHA administration during pregnancy and PROM/pPROM occurrence, which is related in turn to gestational duration and overall fetal health.

## 1. Introduction

Fatty acids (FAs) that contain more than one double bond in their backbone are known as polyunsaturated FAs (PUFAs). The two principal families of PUFAs are ω-3 and ω-6 FAs. They are incorporated in cellular membranes and have important structural and metabolic functions. The essential fatty acids (EFAs), linoleic (LA, 18:2 ω-6) and α-linolenic acid (ALA, 18:3 ω-3), can be converted into other PUFAs such as dihomo-γ-linolenic acid (DGLA, 20:3 ω-6), arachidonic acid (AA, 20:4 ω-6) and eicosapentaenoic acid (EPA, 20:5 ω-3), docosahexaenoic acid (DHA, 22:6 ω-3), respectively. These PUFAs are required to maintain the fluidity, permeability and integrity of cell membranes. AA is the precursor of a family of molecules known as eicosanoids that act as mediators of inflammation, while EPA and DHA are the precursors of resolvins and protectins, that exert anti-inflammatory and protective activities [1,2].

Plasma levels of PUFAs are determined by dietary intake and endogenous metabolism. The human body cannot synthesize EFAs, which, therefore, are obtained from the diet. Increased consumption of vegetable oils and “western diets”, characterized by low fish consumption, lead to a decline of ω-3 PUFAs intake in favour of ω-6 PUFAs and to an altered ω-3/ω-6 FA ratio from 1:10 to 1:(20–25), a so called pro-inflammatory ratio [3]. The optimal ω-3/ω-6 FA ratio is 1:5. This ratio can be obtained by enhancing weekly intake of fish or derivates (≥200 g/week), or with ω-3 FA supplementation [3].

**Figure 1.**
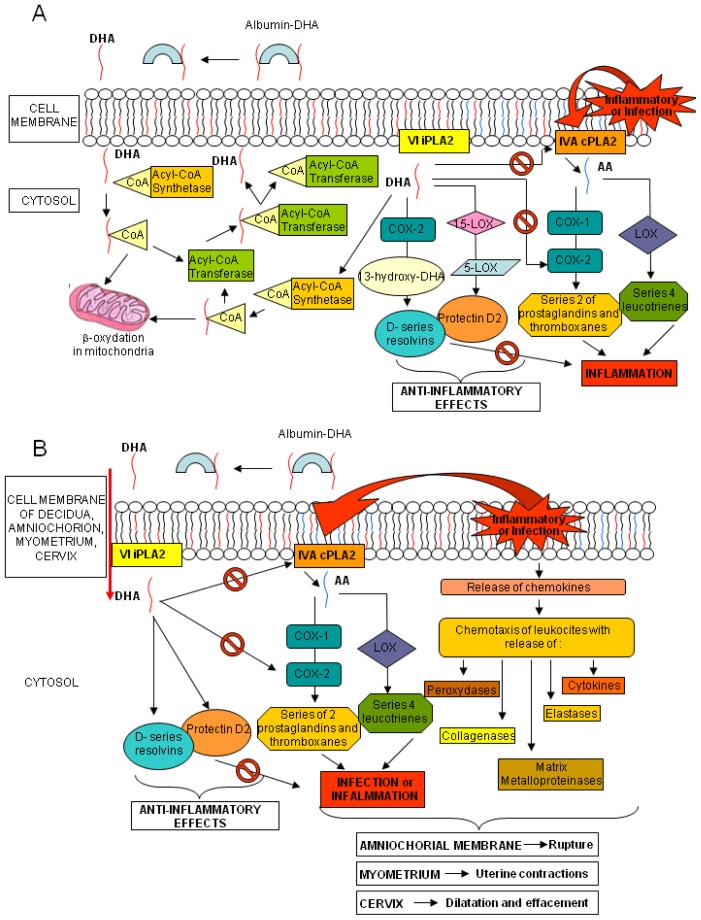
Metabolism of DHA. Panel (**A**) Interactions in DHA and AA metabolic cascades; and Panel (**B**) DHA, resolvin and protectin putative effects on labor and amniochorial membrane rupture events.

Increasing evidence suggests a critical role of-3 PUFAs, and particularly DHA, during healthy pregnancy. According to the World Health Organization (WHO) and the Food and Agriculture Organization of the United Nations (FAO) a daily intake of at least 2.6 g of ω-3 FAs and 100–300 mg of DHA is advisable during pregnancy [4]. A regular consumption of fish or ω-3 FAs supplements during pregnancy results in increased circulating values of ω-3 PUFAs in maternal blood, that is important for both fetal and maternal health [5,6]. Fetal lipogenesis is not completely understood, but the bulk of EFAs seems to derive from the maternal dietary intake, depending on the quantity and composition of maternal PUFAs and on subsequent placental transfer. During gestation, large amounts of DHA are transferred from maternal to fetal blood, hence ensuring high levels of DHA in both placenta and fetal bloodstream and tissues [7]. Fetal DHA demand increases exponentially with gestational age, especially in the third trimester, due to the development of the nervous system and retina [8]. Cord DHA concentration at birth is higher than maternal blood levels, implying high fetal DHA demand in the last months of pregnancy and a preferential placental transfer to the foetus [9]. Mechanisms involved in placental transfer are not entirely known; it seems that placental lipoprotein lipase (LPL), mainly endothelial lipases (EL), hydrolyze maternal triglycerides and phospholipids, increasing the amount of free FA for placental uptake and transfer to the foetus, through passive diffusion or protein-mediated transfer [10,11]. PUFAs are found mainly in phospholipids, especially in sn-2 position, in maternal plasma and erythrocytes and in placental tissue [7,12,13]. The EL of placenta is selective for the phospholipids containing DHA at the sn-2 position, increasing the *in situ* concentration of DHA and its possible transfer to foetus [14]. Several studies have addressed the effects of ω-3 FA supplementation during pregnancy. Higher seafood intake or ω-3 FA supplementation could increase gestational length, decrease incidence of preterm delivery, and positively affect birth weight and newborn health [15–19]. In two clinical trials, ω-3 FA supplementation seemed to enhance pregnancy duration while in other studies on women with high-risk pregnancies, ω-3 FA supplementation appeared to decrease the preterm and early preterm delivery rates [20–23]. Dietary DHA consists of triglycerides or phospholipids. These cannot be absorbed by the intestine, and are broken down into free FAs and then adsorbed and transported in the blood by lipoproteins and albumin. Once the DHA reaches the target tissue, it is released by its carriers, crosses into the cytosol and it is activated by an Acyl-CoA synthetase. Part of the complex DHA-CoA is β-oxidized in mitochondria and a large part is esterified via an Acyl-CoA transferase to the sn-2 position of phospholipids. Phospholipids, containing DHA, can release it by the VI Ca^2+^-independent-phospholipase A_2_ (VI iPLA_2_); part of DHA is afterwards converted into resolvins and protectins by cyclooxygenase-2 (COX-2), 15- and 5-lipoxygenase (15- and 5-LOX, respectively), whereas the remaining is activated by an Acyl-CoA synthetase and mainly re-esterified into the sn-2 position into phospholipids with a little part still available for β-oxidation (Figure 1, panel A) [24]. AA, similarly to DHA, is found in the sn-2 position of membrane phospholipids. After exposure to inflammatory *stimuli* or infections, AA is hydrolyzed from membrane phospholipids by the AA-selective Ca^2+^-dependent cytosolic phospholipase A2 (cPLA2)-IVA (IVA cPLA_2_) and is metabolized to bioactive eicosanoids by COX-1 or COX-2 and LOX. Of the two COX isoenzymes, COX-1 is readily available, whereas COX-2 is present but can also be induced by various *stimuli* [25]. The beneficial role of DHA seems correlated to its direct inhibiting action on the AA and its eicosanoids and to the anti-inflammatory and protective properties of the resolvins and protectins. Indeed, dietary deprivation of DHA in rats is associated with increased expression of IVA cPLA_2_ and COX-2 [26]. Similar mechanisms also occur in human tissues (Figure 1, panel A) [27].

The evidence that labor and rupture of the amniochorial membranes at term, might be caused by the action of cytokines, eicosanoids and enzymes, released by the chorion-decidua, cervix and myometrium [28], prompted us to consider the possible preventive role of DHA in the process of rupture of membrane (ROM) before labor. Both infection and inflammation cascades, through the production of chemokines, recruit leukocytes, releasing peroxidases, elastases, collagenases and matrix metalloproteinases. The same phenomena activate cPLA_2_, starting the AA metabolism with the synthesis of prostaglandins and thromboxanes (series-2) and leukotrienes (series-4). All these mediators have a role in different tissues: in the myometrium they induce uterine contractions, in the cervix they cause effacement and dilation, while in amniochorial membranes, through the degradation of collagen fibers, they may decrease physical resistance causing the rupture. The same mechanisms may could also lead to events such as preterm labor and ROM at any gestational age. The effects of DHA and the anti-inflammatory properties of its mediators could modify the micro-environment of the chorion-decidua, cervix and myometrium, reducing the incidence of these events (Figure 1, panel B).

Another protective effect of DHA on amniochorial membranes is suggested by studies on erythrocyte resistance; indeed, the enrichment of the red cell membrane in ω-3 FAs leads to an increase in the total unsaturation index, erythrocyte resistance to hemolysis, and cell membrane fluidity [29–31]. Supplementation with fish oil increases plasma, membrane, and tissue composition of ω-3 FA, depending on the dose and duration of treatment [32]. Similarly, ω-3 FA can increase the resistance and the stability of membrane of other cells and tissues, such as the amniochorial membrane, making them less prone to rupture. Therefore, an early and optimal supplementation of ω-3 FAs should be guaranteed, during the entire duration of the pregnancy. While the effect of ω-3 FAs supplementation on pregnancy duration and reduction of preterm birth has already been suggested, studies to assess the effect of DHA levels alone on ROM are still lacking [20–23]. Indeed, ROM associated to preterm delivery occurs in approximately 12% of pregnancies, with a significant impact on maternal and fetal morbidity and mortality [33]. In detail, ROM before the labor can be classified into premature rupture of membranes (PROM), that occurs after the 37th week of pregnancy, and preterm-PROM (pPROM) that happens at any earlier gestational age. Incidence of pPROM and PROM is respectively 3% and 10% of all pregnancies [34]. The most significant risk factor for PROM is intrauterine infection, which occurs in 10% of cases. The risk increases with the duration of ROM and affects 40% of patients within 24 h or more from rupture to delivery [35]. However, in a large randomized trial, more than 50% of women with PROM went into labor within 5 h of rupture and about 95% delivered within 28 h of ROM [36]. The gestational age at ROM is crucial. Lower gestational ages mean higher maternal and fetal risks. The maternal *sequelae* associated with pPROM include intrauterine and postpartum infection with possible fetal sepsis. pPROM is associated with preterm delivery with all the complications this entails. A review of randomized trials reported that 76% of patients with pPROM delivered within 1 week, while another study found that 57% of patients delivered within 1 week for pPROM occurring before and around the time of neonatal viability [37,38]. Respiratory distress syndrome, bronchodysplasia, retinopathy prematurity, necrotizing enterocolitis, intraventricular haemorrhage and persistent fetal circulation, are usually associated to pre-term delivery, seriously compromising neonatal health. Umbilical cord compression or prolapse, placental abruption, retained placenta, postpartum hemorrhage, pulmonary hypoplasia and congenital malformations, due to oligohydramnios, antenatal fetal demise and neonatal death are some of the severe consequences of preterm birth.

Based on these premises, we have considered in a small-scale pilot study the effects of DHA supplementation on PUFAs levels during pregnancy and its effect on ROM incidence and gestational duration.

## 2. Pilot Experimental Section

**Figure 2.**
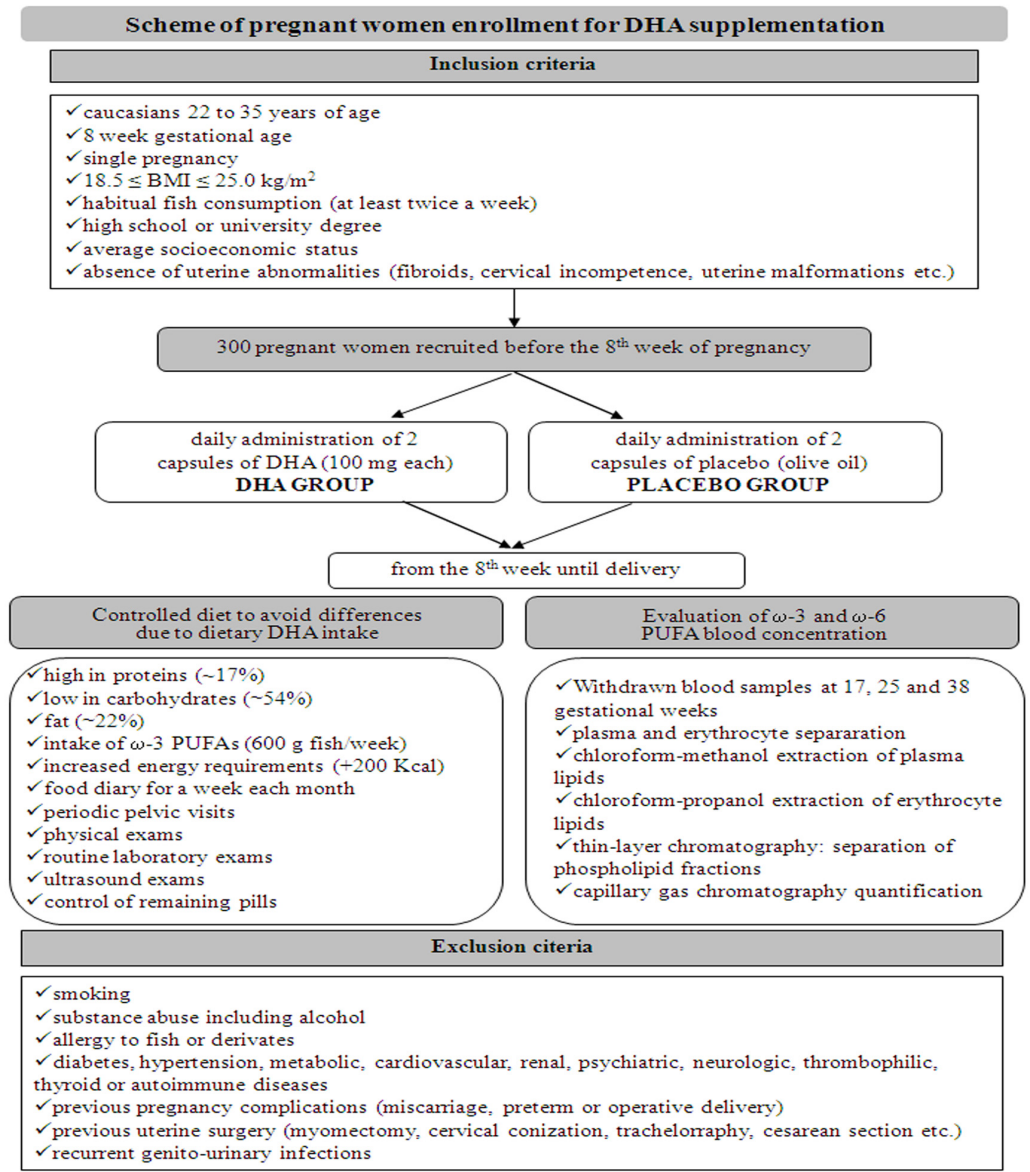
Scheme of pregnant women enrollment: inclusion and exclusion criteria, diet features and laboratory exams for monitoring of DHA supplementation.

Three hundred pregnant women were recruited at the Department of Obstetrics and Gynaecology, San Camillo Forlanini Hospital, Rome, Italy, before the 8th week of pregnancy. In Figure 2 the inclusion/exclusion criteria are reported. We administered DHA, rather than a ω-3 FA supplemented cocktail, to pregnant women who met the criteria (Figure 2, Table 1). The DHA supplementation consisted in 2 capsules of DHA (100 mg each) administered daily (DHA group), *vs.* 2 capsules of placebo (olive oil) to be taken each day (control group), until delivery. In order to avoid differences due to dietary DHA, the women’s diet was checked in composition and energy content requirements. Every woman was invited to fill a food diary each month and to undergo a clinical evaluation and laboratory exams. To evaluate ω-3 and ω-6 PUFA blood concentration, women were submitted at 17, 25, and 38 weeks to specific blood tests, including plasma and erythrocyte fraction separation, and lipid quantification of both fractions by capillary gas chromatography (GC) techniques (Figure 2).

Data are reported as frequencies or percentages for categorical variables, and as mean ± SD for continuous variables. Fisher exact test and the two-tailed unpaired Student’s *t* test were used for dichotomous variables and continuous variables, respectively. The evaluation of differences in membrane rupture between both groups was performed by the χ^2^ test. The data were analyzed using the intention-to-treat principle, that is, values recorded at baseline were compared to values recorded at the end of pregnancy in all patients regardless of treatment duration. A two-tailed *p* value <0.05 was considered statistically significant. Minitab 15 English (Microsoft, 2010) was used for the statistical analysis.

GC determinations revealed increased levels of ω-3 FA and DHA in the maternal blood, through the second and third trimester of pregnancy (Table 2), counteracting the physiological decline of these levels normally observed in the last trimester of pregnancy in untreated mothers [9]. As the dietary intake of ω-3 PUFAs was the same both in study and placebo groups, the significant differences observed between the study groups were probably due to specific DHA supplementation. Furthermore, we observed a reduction of AA levels in erythrocyte and plasma during gestational time, especially in the DHA supplemented group (Table 2). The lower availability of AA and consequently of its eicosanoids, with vasoconstrictional, inflammatory and platelet pro-aggregating activity, could be implicated in placental blood flow increase, with beneficial effects on fetal development and growth. Therefore, the increase of DHA and the decrease AA could play a significant role in guaranteeing normal pregnancy protraction, for their anti-inflammatory properties, leading to increase resistance of amniochorial membranes. A reduced incidence of membrane rupture and a longer duration of pregnancy in the considered DHA-treated group was actually observed, possibly correlating with the DHA supplementation (Table 3).

## 3. Future Perspective

The awareness of maternal, fetal, and neonatal risks associated with membrane rupture before labor and premature delivery makes investigation of DHA protective properties mandatory.

We suggest that maternal and fetal DHA supplementation may result in beneficial effects due to its anti-oxidative and anti-inflammatory properties and might prevent placenta-related disorders [39], but available data are only sparse.

Only recently, the work of Quinlivan and Pakmehr [40], through a pivotal systematic literature review, presented evidence that fish oil supplementation represents a population strategy to reduce early preterm birth. Indeed, fish oils act as competitive antagonists of series-2 prostaglandins, targeting the premature cervical ripening, which is a cause of early preterm birth. Therefore, ω-3 PUFAs dietary supplements offer an exciting avenue for future research, appearing safe and effective in reducing the incidence of early preterm birth. However, adjustments in timing and dosage are required to develop a protocol for routine clinical practice. With this purpose, the authors [40] have started a significant randomised trial ORIP (Omega-3 to reduce the Incidence of Preterm birth), in which 4700 women have been recruited and followed to actually clarify the timing and dose of ω-3 oils. This project is aimed at providing a definitive answer to the question of whether and how maternal fish oil supplementation reduces early preterm birth [40].

Moreover, the ω-3 PUFAs and, in particular, DHA and EPA administration during pregnancy represent not only a potential therapy in pregnancy complications, but also can exert beneficial effects in treating perinatal depressive symptoms [41,42]. Different observational and clinical studies in pregnant women have associated a lower ω-3 FA intake (e.g., fish consumption) with higher rates of anti-depressive therapy up to one year postnatally [42,43], resulting in social behaviour development difficulties of the related infants [42,44]. In addition, FAs supplementation is crucial for the cerebral expansion [45] and neurodevelopmental processes [46,47]. However, Makrides and co-workers [48], have developed a large multicenter, randomized trial in five Australian maternity hospitals to assess whether increasing DHA during the last half of pregnancy reduces depressive symptoms in women and enhances the neurodevelopmental outcome of their children. In this paper 2399 women (gestational age > 21 weeks) were recruited and DHA acid-rich fish oil capsules or matched vegetable oil capsules without DHA were administered. The results showed that the percentage of women with high levels of depressive symptoms did not differ between DHA and control groups (9.67% *vs.* 11.19%) during the first 6 months postpartum. Also, to test the influence of DHA in neonate cognitive and language development, their children were randomly enrolled (*n* = 726) and monitored by the Bayley Scales of Infant and Toddler Development, at 18 months. Interestingly, the results showed that mean cognitive composite scores and mean language composite scores of children in the DHA group did not differ from children in the control group [48].

Recently, a maternal diet with low levels of ω-3 FAs has been observed to predispose infant to allergic disease [49], and it has been suggested that EPA and DHA intake during pregnancy could be related to a reduction of allergic disease risk development in childhood [50,51].

Based on the preliminary data herein reported, large patient cohorts and datasets are now required to shape further whole studies and to assess statistical significance between DHA administration during pregnancy and PROM/pPROM occurrence, and its relation to gestational duration and eventually fetal health. The priority is to investigate the mother-infant couple in its completeness, translating our pilot ideas into well designed trials, framed in the context of advanced nutritional, fully-developed analytical methods, midwifery skills and neonatology competences.

## Figures and Tables

**Table 1.** Pregnant women personal history.

Characteristic	DHA group *n* = 129	Placebo group *n* = 126
Age (years) Mean ± SD	30.86 ± 4.18	29.92 ± 4.80
BMI (Kg/m^2^) Mean ± SD	23.24 ± 1.60	22.65 ± 2.11

**Parity (%)**		

0	46 (36)	50 (40)
1	83 (64)	76 (60)

**Amniocentesis (%)**		

Yes	20 (16)	13 (10)
No	109 (84)	113 (90)

**Genital infection with effective treatment (%)**		

Yes	9 (7)	13 (10)
No	120 (93)	113 (90)

**Table 2.** Erythrocyte and plasma levels of ω-3 and ω-6 FAs at 17, 25 and 38 gestational weeks.

FAs	Erythrocyte FAs (nmol/mL)					Plasma FAs (nmol/mL)				

	DHA group		Placebo group		*p*	DHA group		Placebo group		*p*
	
	Mean	SD	Mean	SD		Mean	SD	Mean	SD	
**17th week**	***n*** **= 129**		***n*** **= 126**			***n*** **= 129**		***n*** **= 126**		

20:3 ω-6 *	126	9.06	127	0.52	0.31	247	15.49	248	15.71	0.40
20:4 ω-6	337	28.47	336	28.58	0.84	237	13.35	235	13.74	0.37
20:5 ω-3	8	4.13	8	4.73	0.14	25	4.95	26	5.48	0.13
22:6 ω-3	149	21.16	148	20.47	0.70	135	7.89	134	7.81	0.41
Total ω-3 FAs	156	21.03	156	21.44	0.95	159	8.54	160	9.62	0.86
Total ω-6 FAs	463	30.40	463	29.41	0.91	483	20.46	483	20.82	0.96
ω-6/ω-3	3.00	0.37	3.02	0.47	0.70	3.04	0.21	3.04	0.21	0.96

**25th week**	***n*** **= 129**		***n*** **= 126**			***n*** **= 129**		***n*** **= 126**		

20:3 ω-6	147	41.08	139	31.93	0.08	338	16.13	341	12.80	0.16
20:4 ω -6	248	12.16	275	10.62	0.00	269	16.72	277	15.95	0.00
20:5 ω-3	11	6.19	11	4.41	0.19	28	7.06	27	7.80	0.27
22:6 ω-3	201	13.10	186	18.12	0.00	220	14.97	189	29.29	0.00
Total ω-3 FAs	213	14.43	196	18.80	0.00	248	17.65	217	31.05	0.00
Total ω-6 FAs	395	46.76	510	33.63	0.00	607	27.38	618	21.89	0.00
ω-6/ω-3	1.86	0.24	2.13	0.27	0.00	2.45	0.19	2.91	0.43	0.00

**38th week**	***n*** **= 124**		***n*** **= 115**			***n*** **= 124**		***n*** **= 115**		

20:3 ω-6	110	11.10	110	11.19	0.96	304	6.36	303	6.85	0.27
20:4 ω -6	224	26.75	252	19.62	0.00	255	18.14	261	15.24	0.01
20:5 ω-3	6	4.18	5	3.41	0.12	31	6.10	30	5.91	0.26
22:6 ω-3	174	12.71	126	17.76	0.00	178	9.04	137	20.58	0.00
Total ω-3 FAs	179	13.98	130	17.96	0.00	209	11.54	167	20.82	0.00
Total ω-6 FAs	334	30.77	362	21.30	0.00	559	20.06	564	18.64	0.04
ω-6/ω-3	1.87	0.22	2.83	0.40	0.00	2.68	0.17	3.43	0.45	0.00

* 20:3 ω-6 Dihomoγlinoleic acid (DGLA); 20:4 ω-6 Arachidonic acid (AA); 20:5 ω-3 Eicosapentaenoic acid (EPA); 22:6 ω-3 Docosahexaenoic acid (DHA).

**Table 3.** Cases of ROM observed during the pivotal study.

ROM	DHA group *n =* 129	Placebo group *n =* 126	Total *n =* 255	*p* value
pPROM	1	4	5	0.02
PROM	5	12	17	0.02

Total (%)	6 (4.7)	16 (1.3)	22 (9)	

## References

[1] Serhan C.N., Chiang N., van Dyke T.E. (2008). Resolving inflammation: Dual anti-inflammatory and proresolution lipid mediators. Nat. Rev. Immunol.

[2] De Caterina R., Basta G. (2001). n-3 Fatty acids and the inflammatory response—Biological background. Eur. Heart J. Suppl.

[3] Simopoulos A.P. (2011). Evolutionary aspects of diet: The omega-6/omega-3 ratio and the brain. Mol. Neurobiol.

[4] Simopoulos A.P., Leaf A., Salem N. (2000). Workshop statement on the essentiality of and recommended dietary intakes for omega-6 and omega-3 fatty acids. Prostaglandins Leukot. Essent. Fatty Acids.

[5] Sanjurjo P., Matorras R., Perteagudo L. (1995). Influence of fatty fish intake during pregnancy in the polyunsaturated fatty acids of erythrocyte phospholipids in the mother at labor and newborn infant. Acta Obstet. Gynecol. Scand.

[6] Otto S.J., van Houwelingen A.C., Hornstra G. (2000). The effect of supplementation with docosahexaenoic and arachidonic acid derived from single cell oils on plasma and erythrocyte fatty acids of pregnant women in the second trimester. Prostaglandins Leukot. Essent. Fatty Acids.

[7] Gil-Sánchez A., Larqué E., Demmelmair H., Acien M.I., Faber F.L., Parrilla J.J., Koletzko B. (2010). Maternal-fetal *in vivo* transfer of [^13^C]docosahexaenoic and other fatty acids across the human placenta 12 h after maternal oral intake. Am. J. Clin. Nutr.

[8] Lauritzen L., Hansen H.S., Jorgensen M.H., Michaelsen K.F. (2001). The essentiality of long chain n-3 fatty acids in relation to development and function of the brain and retina. Prog. Lipid Res.

[9] Montgomery C., Speake B.K., Cameron A., Sattar N., Weaver L.T. (2003). Maternal docosahexaenoic acid supplementation and fetal accretion. Br. J. Nutr.

[10] McCoy M.G., Sun G.S., Marchadier D., Maugeais C., Glick J.M., Rader D.J. (2002). Characterization of the lipolytic activity of endothelial lipase. J. Lipid Res.

[11] Hanebutt F.L., Demmelmair H., Schiessl B., Larque E., Koletzko B. (2008). Long-chain polyunsaturated fatty acid (LC-PUFA) transfer across the placenta. Clin. Nutr.

[12] Parra M.S., Schnaas L., Meydani M., Perroni E., Martínez S., Romieu I. (2002). Erythrocyte cell membrane phospholipid levels compared against reported dietary intakes of polyunsaturated fatty acids in pregnant Mexican women. Public Health Nutr.

[13] Klingler M., Demmelmair H., Larque E., Koletzko B. (2003). Analysis of FA contents in individual lipid fractions from human placental tissue. Lipids.

[14] Chen S., Subbaiah P.V. (2007). Phospholipid and fatty acid specificity of endothelial lipase: Potential role of the enzyme in the delivery of docosahexaenoic acid (DHA) to tissues. Biochim. Biophys. Acta.

[15] Allen K.G., Harris M.A. (2001). The role of n-3 fatty acids in gestation and parturition. Exp. Biol. Med.

[16] Facchinetti F., Fazzio M., Venturini P. (2005). Polyunsaturated fatty acids and risk of preterm delivery. Eur. Rev. Med. Pharmacol. Sci.

[17] Olsen S.F., Osterdal M.L., Salvig J.D., Kesmodel M.U., Henriksen T.B., Hedegaard M., Secher N.J. (2006). Duration of pregnancy in relation to seafood intake during early and mid pregnancy: Prospective cohort. Eur. J. Epidemiol.

[18] Olsen S.F., Secher N.J. (2002). Low consumption of seafood in early pregnancy as a risk factor for preterm delivery: Prospective cohort study. Br. Med. J.

[19] Olsen S.F., Grandjean P., Weihe P., Videro T. (1993). Frequency of seafood intake in pregnancy as a determinant of birth weight: Evidence for a dose dependent relationship. J. Epidemiol. Community Health.

[20] Olsen S.F., Sorensen J.D., Secher N.J., Hedegaard M., Henriksen T.B., Hansen H.S., Grant A. (1992). Randomised controlled trial of effect of fish-oil supplementation on pregnancy duration. Lancet.

[21] Szajewska H., Horvath A., Koletzko B. (2006). Effect of n-3 long-chain polyunsaturated fatty acid supplementation of women with low-risk pregnancies on pregnancy outcomes and growth measures at birth: A meta-analysis of randomized controlled trials. Am. J. Clin. Nutr.

[22] Olsen S.F., Secher N.J., Tabor A., Weber T., Walker J.J., Gluud C. (2000). Randomised clinical trials of fish oil supplementation in high risk pregnancies. BJOG.

[23] Horvath A., Koletzko B., Szajewska H. (2007). Effect of supplementation of women in high-risk pregnancies with long-chain polyunsaturated fatty acids on pregnancy outcomes and growth measures at birth: A meta-analysis of randomized controlled trials. Br. J. Nutr.

[24] Green J.T., Orr S.K., Bazinet R.P. (2008). The emerging role of group VI calcium-independent phospholipase A2 in releasing docosahexaenoic acid from brain phospholipids. J. Lipid Res.

[25] Rao J.S., Rapoport S.I. (2009). Mood-stabilizers target the brain arachidonic acid cascade. Curr. Mol. Pharmacol.

[26] Rao J.S., Ertley R.N., DeMar J.C., Rapoport S.I., Bazinet R.P., Lee H.J. (2007). Dietary n-3 PUFA deprivation alters expression of enzymes of the arachidonic and docosahexaenoic acid cascades in rat frontal cortex. Mol. Psychiatry.

[27] Vincentini O., Quaranta M.G., Viora M., Agostoni C., Silano M. (2011). Docosahexaenoic acid modulates *in vitro* the inflammation of celiac disease in intestinal epithelial cells via the inhibition of cPLA_2_. Clin. Nutr.

[28] Gomez-Lopez N., Laresgoiti-Servitje E., Olson D.M., Estrada-Gutièrrez G., Vadillo-Ortega F. (2010). The role of chemokines in term and premature rupture of the fetal membranes: A review. Biol. Reprod.

[29] Van den Berg J.J.M., de Fouw N.J., Kuypers F.A., Roelofsen B., Houtsmuller U.M.T., Op den Kamp J.A.F. (1991). Increased n-3 polyunsaturated fatty acid content of red blood cells from fish oil-fed rabbits increases *in vitro* lipid peroxidation, but decreases hemolysis. Free Radic. Biol. Med.

[30] Mabile L., Piolot A., Boulet L., Fortin L.J., Doyle N., Rodriguez C., Davignon J., Blache D., Lussier-Cacan S. (2001). Moderate intake of n-3 fatty acids is associated with stable erythrocyte resistance to oxidative stress in hypertriglyceridemic subjects. Am. J. Clin. Nutr.

[31] Hashimoto M., Hossain S., Shimada T., Shido O. (2006). Docosahexaenoic acid-induced protective effect against impaired learning in amyloid β-infused rats is associated with increased synaptosomal membrane fluidity. Clin. Exp. Pharmacol. Physiol.

[32] Palozza P., Sgarlata E., Luberto C., Piccioni E., Anti M., Marra G., Armelao F., Franceschelli P., Bartoli G.M. (1996). n-3 Fatty acids induce oxidative modifications in human erythrocytes depending on dose and duration of dietary supplementation. Am. J. Clin. Nutr.

[33] American College of Obstetricians and Gynecologists (2001). ACOG Practice Bulletin. Assessment of risk factors for preterm birth. Clinical management guidelines for obstetrician-gynecologists. Number 31, October 2001 (Replaces Technical Bulletin number 206, June 1995; Committee Opinion number 172, May 1996 ; Committee Opinion number 187, September 1997; Committee Opinion number 198, February 1998; and Committee Opinion number 251, January 2001. Obstet. Gynecol.

[34] EMedicine from WebMD, Medscape’s Continually Updated Clinical Reference.

[35] Seaward P.G., Hannah M.E., Myhr T.L., Farine D., Ohlsson A., Wang E.E., Haque K., Weston J.A., Hewson S.A., Ohel G. (1997). International multicentre term prelabor rupture of membranes study: Evaluation of predictors of clinical chorioamnionitis and postpartum fever in patients with prelabor rupture of membranes at term. Am. J. Obstet. Gynecol.

[36] Hannah M.E., Ohlsson A., Farine D., Hewson S.A., Hodnett E.D., Myhr T.L., Wang E.E., Weston J.A., Willan A.R. (1996). Induction of labor compared with expectant management for prelabor rupture of the membranes at term. N. Engl. J. Med.

[37] Mercer B.M., Arheart K.L. (1995). Antimicrobial therapy in expectant management of preterm premature rupture of the membranes. Lancet.

[38] Schucker J.L., Mercer B.M. (1996). Midtrimester premature rupture of the membranes. Semin. Perinatol.

[39] Jones M.L., Mark P.J., Waddell B.J. (2014). Maternal dietary omega-3 fatty acids and placental function. Reproduction.

[40] Quinlivan J.A., Pakmehr S. (2013). Fish oils as a population based strategy to reduce early preterm birth. Reprod. Syst. Sex. Disord.

[41] Mozurkewich E.L., Clinton C.M., Chilimigras J.L., Hamilton S.E., Allbaugh L.J., Berman D.R., Marcus S.M., Romero V.C., Treadwell M.C., Keeton K.L. (2013). The Mothers, Omega-3, and Mental Health Study: A double-blind, randomized controlled trial. Am. J. Obstet. Gynecol.

[42] Deligiannidis K.M., Freeman M.P. (2014). Complementary and alternative medicine therapies for perinatal depression. Best Pract. Res. Clin. Obstet. Gynaecol.

[43] Strøm M., Mortensen E.L., Halldorsson T.I., Thorsdottir I., Olsen S.F. (2009). Fish and long-chain n-3 polyunsaturated fatty acid intakes during pregnancy and risk of postpartum depression: A prospective study based on a large national birth cohort. Am. J. Clin. Nutr.

[44] Skotheim S., Braarud H.C., Høie K., Markhus M.W., Malde M.K., Graff I.E., Berle J.Ø., Stormark K.M. (2013). Subclinical levels of maternal depression and infant sensitivity to social contingency. Infant Behav. Dev.

[45] Crawford M.A., Broadhurst C.L. (2012). The role of docosahexaenoic and the marine food web as determinants of evolution and hominid brain development: The challenge for human sustainability. Nutr. Health.

[46] Loomans E.M., van den Bergh B.R., Schelling M., Vrijkotte T.G., van Eijsden M. (2014). Maternal long-chain polyunsaturated fatty acid status during early pregnancy and children’s risk of problem behavior at age 5–6 years. J. Pediatr.

[47] Rogers L.K., Valentine C.J., Keim S.A. (2013). DHA supplementation: Current implications in pregnancy and childhood. Pharmacol. Res.

[48] Makrides M., Gibson R.A., McPhee A.J., Yelland L., Quinlivan J., Ryan P., DOMInO Investigative Team (2010). Effect of DHA supplementation during pregnancy on maternal depression and neurodevelopment of young children: A randomized controlled trial. JAMA.

[49] Lumia M., Luukkainen P., Tapanainen H., Kaila M., Erkkola M., Uusitalo L., Niinistö S., Kenward M.G., Ilonen J., Simell O. (2011). Dietary fatty acid composition during pregnancy and the risk of asthma in the offspring. Pediatr. Allergy Immunol.

[50] Pistiner M., Gold D.R., Abdulkerim H., Hoffman E., Celedon J.C. (2008). Birth by cesarean section, allergic rhinitis, and allergic sensitization among children with a parental history of atopy. J. Allergy Clin. Immunol.

[51] Makrides M., Gunaratne A.W., Collins C.T. (2013). Dietary n-3 LC-PUFA during the perinatal period as a strategy to minimize childhood allergic disease. Nestle Nutr. Inst. Workshop Ser.

